# Effects of seasonal variations and meteorological factors on IVF pregnancy outcomes: a cohort study from Henan Province, China

**DOI:** 10.1186/s12958-022-00986-3

**Published:** 2022-08-06

**Authors:** Ting Chu, Di Wang, Ting Yu, Jun Zhai

**Affiliations:** 1 Center for Reproductive Medicine, The First Affiliated Hospital of Zhengzhou University, Zhengzhou, China; 2 Henan Provincial Obstetrical and Gynecological Diseases (Reproductive Medicine) Clinical Research Center, The First Affiliated Hospital of Zhengzhou University, Zhengzhou, China

**Keywords:** IVF, Season, Temperature, Embryonic development, Pregnancy outcome

## Abstract

**Objective:**

To investigate whether seasonal variations and meteorological factors influence pregnancy outcomes in women undergoing in vitro fertilization-embryo transfer (IVF-ET) treatment.

**Design:**

Retrospective cohort study.

**Setting:**

University-affiliated reproductive medical center.

**Subjects:**

Women aged < 35 years undergoing IVF from June 1, 2015, to June 1, 2019.

**Interventions:**

Cycles were divided into four groups according to the date of the beginning of ovulation induction: spring (659 cycles), summer (578 cycles), autumn (519 cycles), and winter (534 cycles).

**Results:**

The high-quality embryo rate was higher in autumn and winter than in cycles in which ovulation induction occurred in spring and summer (58.70% vs. 58.78% vs. 62.67% vs. 63.42%; *P* < 0.001). The results of linear regression analysis showed that the high-quality embryo rate was significantly correlated with the daily average temperature of ovulation induction (*P* = 0.037). The clinical pregnancy rates of cycles starting ovulation induction in spring, summer, and autumn were significantly higher than those starting in winter (70.71% vs. 73.18% vs. 70.13% vs. 65.17%; *P* = 0.031), while the biochemical pregnancy rate, early abortion rate, and live birth rate were not significantly different (*P* > 0.050). Multivariate logistic regression analysis showed significant seasonal variation in clinical pregnancy (OR = 1.643, 95% CI = 1.203–2.243; *P* = 0.002), and that a higher daily average temperature at the time of ovulation induction increased the clinical pregnancy rate (OR = 1.012, 95% CI = 1.001–1.022; *P* = 0.031).

**Conclusions:**

In women younger than 35 years who undergo IVF treatment, the season and ambient temperature on the date of the beginning of ovulation induction may have an impact on embryo development and clinical pregnancy.

## Introduction

Spontaneous conception has seasonal variations, and multiple pregnancies, ectopic pregnancy, spontaneous abortion, stillbirth, and live birth are all thought to be impacted by seasonal variations [1–3]. Studies have found that among naturally conceived pregnancies in the USA, birth rates are higher in summer and early autumn (July to September) than in spring (April and May), whereas in Northern Europe, birth rates are highest in spring (March and April) and lowest in autumn (October and November) [4]. Natural conception may be influenced by a number of factors, including cultural and sociological factors, as well as environmental factors.

Assisted reproductive technology (ART) provides a good model for investigating the effects of meteorological changes on a woman's conception process, because the patient's physiological status and meteorological factors can be more easily determined [5]. Although many studies of seasonal variation and meteorological factors on pregnancy outcomes in women who underwent in vitro fertilization (IVF) treatment have been conducted in identical climatic conditions, their results are varied [5–8]. A study conducted in Jerusalem showed that the quality of the embryo and fertilization rates were affected by seasonal variation during ART treatment [5], whereas another study found that pregnancy outcomes of ART did not follow any specific seasonal variation [6]. Omsk, Russia, and Alberta, Canada, have a similar humid continental climate; however, studies from Omsk identified higher pregnancy rates in summer and autumn [7], whereas studies from Alberta found no significant seasonal variation in pregnancy outcomes [8]. The differences in the results may be contributed to the differences in the study population and the geographical environment in which the study population is located, and the criteria for assigning patients to the corresponding seasons are inconsistent in various studies (e.g., according to the day of stimulation, oocyte retrieval, or embryo transfer).

Therefore, we conducted a large cohort study of more than 2000 IVF cycles at a reproductive medical center to investigate the effects of seasonal and meteorological factors on the date of the beginning of ovulation induction on embryo development and pregnancy outcomes in women who had undergone IVF treatment. Multivariate logistic regression analysis was performed to understand the seasonal factors that are associated with IVF pregnancy outcomes for improved clinical application.

## Materials and methods

### Study design

We conducted a retrospective cohort study of women who underwent IVF for the first time at the Reproductive Medicine Center of the First Affiliated Hospital of Zhengzhou University from June 1, 2015, to June 1, 2019. All patients were treated using the follicular phase long-acting long protocol. And only 2290 women had a successful first fresh embryo transfer cycle that met the inclusion and exclusion criteria. Henan Province has a warm temperate subtropical monsoon climate. All cycles were divided into spring (March–May), summer (June–August), autumn (September–November), and winter (December–February) according to the date of the beginning of ovulation induction.

The inclusion criteria were as follows: 1) age 20–35 years, to eliminate the effect of advanced age on pregnancy outcomes, 2) normal ovarian function (antral follicle count [AFC] > 7, anti-Mullerian hormone [AMH] > 1.1 ng/mL), 3) first fresh IVF cycle using the follicular phase long-term protocol for ovulation induction, and 4) fresh embryo transfer after oocyte retrieval. The exclusion criteria were as follows: 1) cycle cancellation of fresh embryo transfer due to liver or kidney dysfunction, pre-implantation genetic diagnosis/pre-implantation genetic screening, or personal reasons; 2) a history of endometriosis, adenomyosis, uterine malformation, endometrial polyps, uterine fibroids, scarred uterus, pelvic tuberculosis, cervical insufficiency, cervical conization, severe hydrosalpinx, polycystic ovary syndrome, repeated implantation failure, recurrent miscarriages, or endocrine diseases; 3) chromosomal abnormalities (for either the woman or man, or both); and 4) severe oligozoospermia or teratozoospermia in the male partner.

This study was approved by the Ethics Committee for Scientific Research and Clinical Trials of the First Affiliated Hospital of Zhengzhou University. Written informed consent for participation was not required for this study, in accordance with the national legislation and institutional requirements.

### Meteorological data

The meteorological data for June 1, 2015, to June 1, 2019, for Zhengzhou City, Henan Province, China were downloaded from the China Meteorological Data Network (http://data.cma.cn/). The data included daily average temperature, daily average humidity, and sunshine duration.

### IVF protocol

#### Downregulation regimen

On day 2–3 of the menstrual cycle, patients were given 3.75 mg of a long-acting gonadotropin (Gn)-releasing hormone agonist (Diphereline, 3.75 mg; Beaufour-Ipsen, Dreux, France), by subcutaneous injection, to achieve pituitary downregulation. After 30–42 days, vaginal ultrasound and serum follicle stimulating hormone (FSH), luteinizing hormone (LH), estrogen (E2), and progesterone (P) levels were used to assess pituitary downregulation, and to ensure that the downregulating standard was reached (no functional cyst with diameter > 10 mm, a serum FSH level < 5 IU/L, and LH level < 3 IU/L); controlled ovarian hyperstimulation was subsequently performed.

#### Ovulation induction and oocyte retrieval

The dosage of Gn (GONAL-f; Merck Serono, Darmstadt, Germany) was individualized based on the patient's age, AMH level, AFC, body mass index (BMI), and serum basal FSH level. The dosage of Gn and the addition of human menopausal Gn (LeBold, Zhuhai Livzon Pharmaceutical, China) was considered based on the follicle size and hormone levels. When one dominant follicle was ≥ 20 mm in diameter and at least three dominant follicles were ≥ 17 mm in diameter, we administered 250 mg of Azer (Merck Serono, Italy) and 2000 IU of human chorionic Gn (hCG) (Zhuhai Livzon Pharmaceutical, China). Oocyte retrieval was performed under vaginal ultrasound guidance 36–37 h after the trigger injection.

### Embryo culture and transfer

Fertilization was observed on the first day after oocyte retrieval; double pronucleus (2PN) was considered to identify normal zygotes, and these were transferred into cleavage fluid to continue culture. Cleavage and development of embryos were observed on the third day; embryo quality was assessed according to the number, diameter, morphology, and developmental rate of blastomeres. Fresh embryo transfer was performed according to embryo quality, endometrial status, and patient conditions.

### Outcome measures

The primary outcomes were clinical pregnancy and live birth, and the secondary outcomes were biochemical pregnancy and early abortion. Clinical pregnancy was defined as a pregnancy diagnosed by ultrasonographic visualization of one or more gestational sacs or definitive clinical signs of pregnancy. Live birth was defined as the complete expulsion or extraction from its mother of a product of fertilization, irrespective of the duration of the pregnancy which, after such separation, breathes or shows any other evidence of life, such as heart beat, umbilical cord pulsation, or definite movement of voluntary muscles, irrespective of whether the umbilical cord has been cut or the placenta is attached. Biochemical pregnancy was defined as a pregnancy diagnosed only by the detection of hCG in serum or urine and that does not develop into a clinical pregnancy. Early abortion was defined as the spontaneous loss of a clinical pregnancy occurring before 12 completed weeks of gestational age [9].

Evaluation measures were as follows: 1) ovarian response indicators: total dosage of Gn used, length of Gn used, and number of oocytes retrieved; 2) embryo quality indicators: number of 2PN fertilization, 2PN fertilization rate (number of 2PN fertilization/number of oocytes retrieved × 100), number of 2PN cleavage, 2PN cleavage rate (number of 2PN cleavage/number of 2PN fertilization × 100), number of high-quality embryos, high-quality embryo rate (number of high-quality embryos/number of 2PN cleavage × 100), and number of transferred embryos; 3) pregnancy outcomes: biochemical pregnancy rate (number of hCG positive cycles/number of transplant cycles × 100), clinical pregnancy rate (number of clinical pregnancy cycles/number of transplant cycles × 100), early abortion rate (number of cycles with early abortion/number of cycles with clinical pregnancy × 100), and live birth rate (number of deliveries that resulted in at least one live born baby/number of transplant cycles × 100).

### Statistical analysis

SPSS 22.0 (IBM Corporation, Armonk, NY, USA) was used for data processing and analysis. Measurement data were suggested to meet the normal distribution using the Shapiro–Wilk test; therefore, the measurement data were expressed as mean ± standard deviation. For comparison of continuous variables between multiple groups and when the variance was homogeneous among groups, one-way ANOVA was used. The LSD-t test was used for paired comparisons of continuous variables within the groups. Enumeration data are expressed as the constituent ratio or rate (%). The chi-square test was used to compare differences between categorical variables, and Bonferroni correction was used to account for multiple testing. Linear regression analysis was used for continuous variables, and multivariate logistic regression analysis was used for dichotomous variables. Analysis items with *P* < 0.050 was considered statistically significant.

### Sensitivity analysis

We conducted sensitivity analyses stratified by the day of embryo transfer to verify the stability of our findings. At our center, most fresh embryo transfers occurred at the cleavage stage on Day 3. Day 3 and Day 5 embryo transfers were analyzed separately to assess whether the potential effects of seasonal variation and meteorological factors remained consistent across studies.

## Results

### Patients’ general characteristics

According to the inclusion and exclusion criteria, 2290 patients (2290 fresh IVF cycles) were included in this study. We divided the patients into four groups according to the seasons: 659 cycles in the spring group, 578 cycles in the summer group, 519 cycles in the autumn group, and 534 cycles in the winter group. The results showed that there were no significant differences in age, type of infertility, duration of infertility, infertility factor, BMI, basal E2 level, basal FSH level, basal LH level, basal AMH level, or AFC among patients in different seasons (*P* > 0.050; Table 1).

**Table 1:** Patients’ general characteristics

Item	Spring	Summer	Autumn	Winter	F/ χ2	*P* value
Cycle number	659	578	519	534		
Age (year)	29.00 ± 2.98	28.88 ± 2.99	28.76 ± 2.97	29.07 ± 2.99	1.142	0.331
Type of infertility					1.710	0.635
Primary infertility (%)	43.70% (288/659)	41.18% (238/578)	40.08% (208/519)	41.95% (224/534)		
Secondary infertility (%)	56.30% (371/659)	58.82% (340/578)	59.92% (311/519)	58.05% (310/534)		
Duration of infertility (year)	3.21 ± 2.41	3.11 ± 2.37	2.96 ± 2.20	2.92 ± 2.26	2.057	0.104
Infertility factor						
Tubal factor (%)	93.78% (618/659)	94.29% (545/578)	93.45% (485/519)	93.63% (500/534)	0.375	0.945
Ovulatory dysfunction (%)	4.25% (28/659)	4.15% (24/578)	4.43% (23/519)	4.87% (26/534)	0.399	0.940
Unexplained (%)	1.97% (13/659)	1.56% (9/578)	2.12% (11/519)	1.50% (8.534)	0.882	0.830
BMI (kg/m^2^)	22.48 ± 2.42	22.39 ± 2.33	22.31 ± 2.29	22.49 ± 2.36	0.672	0.569
Basal E2 level (pg/ml)	38.37 ± 18.41	38.94 ± 18.60	38.82 ± 23.79	38.20 ± 23.84	0.160	0.923
Basal FSH level (mIU/ml)	6.65 ± 1.12	6.73 ± 1.15	6.82 ± 1.12	6.77 ± 1.16	2.253	0.080
Basal LH level (mIU/ml)	5.19 ± 2.40	5.33 ± 2.61	5.38 ± 2.93	5.05 ± 2.56	1.744	0.156
Basal AMH level (ng/ml)	3.30 ± 1.92	3.53 ± 2.22	3.62 ± 2.13	3.31 ± 1.85	2.459	0.061
AFC (n)	14.97 ± 5.28	14.96 ± 5.36	15.08 ± 5.42	14.55 ± 5.33	0.996	0.394

*BMI* body mass index, *E2* estradiol, *FSH* follicle-stimulating hormone, *LH* luteinizing hormone, *AMH* anti-Mullerian hormone, *AFC* antral follicle count

### Laboratory results and pregnancy outcomes

**Fig. 1:**
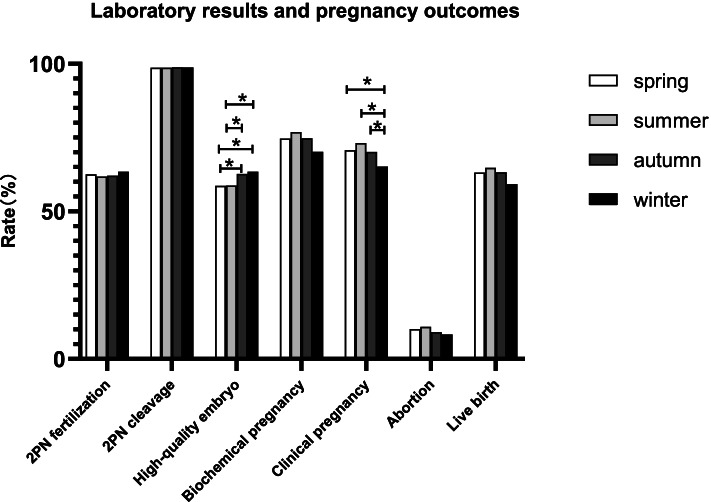
Laboratory results and pregnancy outcomes. Note: * Bonferroni correction, *P* < 0.050

There were no statistically significant differences in the total dosage of Gn used, length of Gn used, number of oocytes retrieved, number of 2PN fertilization, 2PN fertilization rate, number of 2PN cleavage, 2PN cleavage rate, number of high-quality embryos, and number of transferred embryos in each season (*P* > 0.050). The high-quality embryo rate in the autumn and winter groups was significantly higher than that in the spring and summer groups (62.67% vs. 63.42% vs. 58.70% vs. 58.78%; *P* < 0.001). In addition, the clinical pregnancy rate was higher in the spring and summer groups than that in the winter group (70.71% vs. 73.18% vs. 70.13% vs. 65.17%; *P* = 0.031). The biochemical pregnancy rate, early abortion rate, and live birth rate in the winter group were lower than those in the other three groups, but the differences were not statistically significant (*P* > 0.050; Table 2 and Fig. 1).

**Table 2:** Laboratory results and pregnancy outcomes

Item	Spring	Summer	Autumn	Winter	F/ χ2	*P* value
Cycle number	659	578	519	534		
Length of Gn used (d)	13.51 ± 1.95	13.72 ± 1.98	13.71 ± 1.96	13.49 ± 2.06	2.261	0.079
Total dosage of Gn used (IU)	2380.17 ± 858.13	2431.47 ± 821.74	2419.98 ± 835.10	2444.82 ± 823.40	0.683	0.563
Number of oocytes retrieved (n)	13.72 ± 5.09	13.98 ± 5.74	13.85 ± 5.44	13.37 ± 4.92	1.346	0.258
Number of 2PN fertilization (n)	8.56 ± 3.88	8.63 ± 4.14	8.60 ± 3.99	8.49 ± 3.95	0.133	0.940
2PN fertilization rate (%)	62.54% (5644/9042)	61.75% (4990/8081)	62.05% (4461/7189)	63.48% (4532/7139)	5.390	0.145
Number of 2PN cleavage (n)	8.45 ± 3.86	8.52 ± 4.11	8.49 ± 3.95	8.38 ± 3.91	0.129	0.943
2PN cleavage rate (%)	98.71% (5571/5644)	98.70% (4925/4990)	98.79% (4407/4461)	98.74% (4475/4532)	0.198	0.978
Number of high-quality embryos (n)	5.19 ± 3.02	5.01 ± 3.13	5.32 ± 2.97	5.31 ± 2.95	2.345	0.071
High-quality embryo rate (%)	58.70%^c,d^ (3270/5571)	58.78%^c,d^ (2895/4925)	62.67%^a,b^ (2762/4407)	63.42%^a,b^ (2838/4475)	18.031	0.000
Number of transferred embryos (n)	1.83 ± 0.38	1.78 ± 0.41	1.83 ± 0.38	1.80 ± 0.40	1.721	0.161
Day of transfer					6.986	0.072
Day 3 (%)	84.83% (559/659)	79.41% (459/578)	83.62% (434/519)	81.65% (436/534)		
Day 5 (%)	15.17% (100/659)	20.59% (119/578)	16.38% (85/519)	18.35% (98/534)		
Biochemical pregnancy rate (%)	74.66% (492/659)	76.82% (444/578)	74.76% (388/519)	70.22% (375/534)	6.631	0.085
Clinical pregnancy rate (%)	70.71%^d^ (466/659)	73.18%^d^ (423/578)	70.13%^d^ (364/519)	65.17% (348/534)	8.866	0.031
Early abortion rate (%)	10.09% (47/466)	10.87% (46/423)	9.07% (33/364)	8.33% (29/348)	1.657	0.647
Live birth rate (%)	63.13% (416/659)	64.71% (374/578)	63.20% (328/519)	59.18% (316/534)	3.927	0.270

Continuous data, mean ± SD; categorical data, % (n/N)

*Gn* gonadotropin, *d* days, *2PN* 2 pronuclei

^a^Significantly different to spring

^b^Significantly different to summer

^c^Significantly different to autumn

^d^Significantly different to winter

### Logistic regression assessment of pregnancy outcomes

We performed univariate logistic regression analysis on pregnancy outcome indicators (biochemical pregnancy, clinical pregnancy, early abortion, and live birth), in which statistically significant indicators were included in the multivariate logistic regression analysis. After correction for confounding factors, the results showed that the clinical pregnancy rates were 1.253-fold, 1.643-fold, and 1.190-fold higher in the spring, summer, and autumn groups, respectively, compared to the winter group. In addition, we found a significant correlation between the daily average temperature of ovulation induction and clinical pregnancy (odds ratio [OR] = 1.012, 95% CI = 1.001–1.022; *P* = 0.031; Table 3).

**Table 3:** Logistic regression assessment of pregnancy outcomes

	**Biochemical pregnancy rate**			**Clinical pregnancy rate**			**Early abortion rate**			**Live birth rate**		
	***P***	**OR**	**95% CI**	***P***	**OR**	**95% CI**	***P***	**OR**	**95% CI**	***P***	**OR**	**95% CI**
Season		0.115			0.019			0.564			0.189	
Winter		1.00	(Reference)		1.00	(Reference)		1.00	(Reference)		1.00	(Reference)
Spring	0.313	1.173	(0.860,1.598)	0.135	1.253	(1.932,1.684)	0.413	1.217	(0.761,1.946)	0.131	1.199	(0.948,1.517)
Summer	0.016	1.495	(1.078,2.072)	0.002	1.643	(1.203,2.243)	0.179	1.382	(0.862,2.217)	0.052	1.273	(0.998,1.624)
Autumn	0.369	1.163	(0.836,1.618)	0.276	1.190	(0.870,1.627)	0.713	1.100	(0.663,1.824)	0.196	1.179	(0.919,1.512)
Meteorological factors												
Daily average temperature (°C)	0.133	1.008	(0.997,1.020)	0.031	1.012	(1.001,1.022)	0.226	1.010	(0.994,1.027)	0.142	1.006	(0.998,1.015)
Daily average humidity (%)	0.619	1.001	(0.996,1.007)	0.228	1.003	(0.998,1.009)	0.430	1.003	(0.995,1.012)	0.462	1.002	(0.997,1.006)
Sunshine duration (hr)	0.961	0.999	(0.973,1.027)	0.270	0.986	(0.961,1.011)	0.945	0.999	(0.960,1.038)	0.198	0.987	(0.967,1.007)

Reference, this variable functions as an indicator

Biochemical pregnancy adjusted by basal luteinizing hormone level, basal anti-Mullerian hormone (AMH) level, antral follicle count, total dosage of gonadotropin (Gn) used, number of 2PN fertilization, number of 2PN cleavage; clinical pregnancy adjusted by duration of infertility, basal AMH level, total dosage of Gn used, and number of 2PN cleavage; early abortion adjusted by duration of infertility; live birth adjusted by age and duration of infertility

*OR* odds ratio, *CI* confidence interval, *hr* hours

### Linear regression assessment of high-quality embryo rate

There was a significant difference in the high-quality embryo rate among the seasons, and linear regression analysis of the relationship between the high-quality embryo rate and meteorological factors showed that the high-quality embryo rate was significantly correlated with the daily average temperature at ovulation induction (*P* = 0.037; Table 4).

**Table 4:** Linear regression assessment of high-quality embryo rate

	***P* value**	**OR**	**95% CI**	
			**Lower limit**	**Upper limit**
Meteorological factors				
Daily average temperature (°C)	0.037	-0.012	-0.023	-0.001
Daily average humidity (%)	0.056	-0.001	-0.001	0.000
Sunshine duration (hr)	0.319	-0.002	-0.006	0.002

High-quality embryo rate adjusted by age, duration of infertility, body mass index, basal E2 level, basal follicle stimulating hormone level, basal luteinizing hormone level, basal anti-Mullerian hormone level, antral follicle count, length of gonadotropin (Gn) used, total dosage of Gn used, and number of oocytes retrieved

### Sensitivity analyses

In the sensitivity analyses restricted to Day 3 fresh embryo transfers (1888 cycles), the association between seasonal parameters and clinical pregnancy was consistent with the results described in our overall analysis. The clinical pregnancy rate was 52.1% higher (OR = 1.521, 95% CI = 1.064–2.174; *P* = 0.021) in summer compared with cycles starting ovulation induction in winter, and increased with the daily average temperature at ovulation induction (OR = 1.014, 95% CI = 1.001–1.029; *P* = 0.042; Table 5). In cycles with fresh embryo transfer on Day 5 (402 cycles), the ORs for seasonal parameters and clinical pregnancy varied more because of the small sample size.

**Table 5:** Sensitivity analyses

	**Biochemical pregnancy rate**			**Clinical pregnancy rate**			**Early abortion rate**			**Live birth rate**		
	***P***	**OR**	**95% CI**	***P***	**OR**	**95% CI**	***P***	**OR**	**95% CI**	***P***	**OR**	**95% CI**
Season		0.319			0.027			0.802			0.195	
Winter		1.00	(Reference)		1.00	(Reference)		1.00	(Reference)		1.00	(Reference)
Spring	0.392	1.168	(0.819,1.666)	0.146	1.285	(0.917,1.800)	0.513	1.186	(0.711,1.980)	0.084	1.259	(0.969,1.636)
Summer	0.142	1.323	(0.910,1.924)	0.021	1.521	(1.064,2.174)	0.427	1.239	(0.730,2.105)	0.046	1.323	(1.005,1.741)
Autumn	0.877	0.971	(0.671,1.405)	0.780	1.051	(0.740,1.495)	0.963	1.014	(0.578,1.778)	0.318	1.151	(0.873,1.519)
Meteorological factors												
Daily average temperature (°C) Daily average humidity (%) Sunshine duration (hr)	0.464 0.901 0.968	1.005 0.999 0.999	(0.991,1.020) (0.992,1.008) (0.960,1.040)	0.042 0.742 0.228	1.014 0.999 0.977	(1.001,1.029) (0.991,1.006) (0.940,1.015)	0.170 0.271 0.632	1.019 1.008 1.018	(0.992,1.047) (0.994,1.023) (0.947,1.093)	0.165 0.359 0.145	1.009 0.997 0.973	(0.996,1.023) (0.989,1.004) (0.938,1.009)

Reference, this variable functions as an indicator

Biochemical pregnancy adjusted by basal anti-Mullerian hormone (AMH) level, total dosage of gonadotropin (Gn) used, number of 2PN fertilization, number of 2PN cleavage; clinical pregnancy adjusted by basal AMH level, total dosage of Gn used, number of 2PN fertilization, and number of 2PN cleavage; early abortion adjusted by duration of infertility; live birth adjusted by age, duration of infertility, total dosage of Gn used, number of 2PN fertilization, and number of 2PN cleavage

## Discussion

Due to many factors, such as environment, lifestyle, and age at the time of reproduction, the incidence of infertility shows a continuous increasing trend. The emergence of ART has brought hope for fertility to many infertile families [10]. The aim of this study was to investigate whether seasonal variations and meteorological factors affect embryo development and pregnancy outcomes in women undergoing IVF for the first time. Our findings suggest that season and environmental temperature on the date of the beginning of ovulation induction was an important factor affecting embryo development and clinical pregnancy. The clinical pregnancy rate was significantly higher in seasons with higher daily mean temperatures (spring, summer, and autumn) than in winter, which has a lower daily mean temperature, while other environmental factors such as humidity and sunshine duration had no effect on pregnancy outcomes.

There are many controversies about whether seasonal variation and environmental factors are related to pregnancy outcomes in women undergoing IVF treatment. The conclusions drawn from this study are in agreement with previous findings [11, 12]. A study conducted at the University of Arizona, USA, showed that the implantation rate, clinical pregnancy rate, and live birth rate were higher in women who obtained oocytes for ART in summer than in other seasons [11]. A study conducted in Hong Kong, China, also concluded that daily temperature is the most important factor affecting pregnancy outcomes, whereas humidity, sunlight duration, and solar radiation had no effect on pregnancy outcomes [12]. In addition, there are many inconsistent conclusions that pregnancy outcomes in women undergoing IVF treatment are not affected by seasonal variations [6, 8, 13]. On the one hand, it may be because of differences in the geographical environment in which the study populations were located, as well as the use of controlled ovarian hyperstimulation protocols. On the other hand, the time nodes of included patients are also inconsistent. Some studies are based on the date of the beginning of ovulation induction, while others are based on the date of oocyte retrieval or embryo transfer. The conclusions drawn from different study settings are not comparable.

In this study, there was no significant difference in the total dosage of Gn used, length of Gn used, or number of oocytes retrieved among seasons, which is consistent with the results of Wunder et al. [14], indicating that seasonal variations do not affect the ovarian response. The present study showed that the high-quality embryo rate was significantly higher in autumn and winter than in spring and summer, which is consistent with the conclusions of Stolwijk et al. [15], but in contrast to the conclusions of Rojansky et al. [5]. The effect of seasonal variations on human fertility mainly occurs through seasonal variations in daily temperature and light intensity [16]. Photoperiodism is the main environmental factor that causes seasonal variations in mammalian reproduction [17]. Melatonin secretion from the pineal gland is affected by periodic changes in light intensity due to seasonal variations, changing the secretion rhythm of Gn-releasing hormone, which affects multiple aspects such as Gn secretion and follicular development [18]. Melatonin has been shown to improve pregnancy outcomes in IVF by increasing the number of mature oocytes, fertilization rate, and the number of high-quality embryos [10, 19]. The duration of sunshine in autumn and winter can lead to increased melatonin secretion [20, 21], which is capable of improving egg quality. Dragojevic et al. found that melatonin receptors are present in both the central nervous system and peripheral tissues where they play a physiological role [22]. In the present study, all patients used Gn-releasing hormone agonists for hypothalamic and pituitary down-regulation; thus, the effect of light on the hypothalamus and pituitary was diminished and melatonin may act mainly through peripheral tissues.

Although the high-quality embryo rate in autumn and winter was higher than that in spring and summer, our study results showed that the clinical pregnancy rate in winter was lower than that in the other three seasons, which may be related to the vitamin D content in the body. While diet contributes to circulating vitamin D levels, the majority is derived from sun exposure [23]. Vitamin D deficiency and secondary hyperparathyroidism are more likely to occur in winter than in summer [24]. Some studies have shown no association between vitamin D levels and clinical outcomes of IVF [25–27]. Howover, a meta-analysis of 11 published cohort studies concluded that adequate vitamin D levels are associated with higher clinical pregnancy and live birth rates among women undergoing ART [28]. In addition, factors other than season, temperature, and vitamin D levels may also be involved. For example, lifestyle changes associated with diet and activity in warm months may lead to better clinical outcomes during these months [29]. Future research should consider including the personal factors of couples that may be influenced by seasonal factors.

It is noteworthy that this study has many strengths. First, to reduce the effect of age on pregnancy outcomes, the included sample was limited to women aged < 35 years. Second, Zhengzhou City, Henan Province belongs to a continental monsoon climate with moderate cold and warm temperatures and four clear seasons, which provides favorable environmental conditions for studying the effects of seasonal factors on pregnancy outcomes. Furthermore, this study is the first to focus on a population undergoing ovulation induction using the follicular phase long-acting long protocol, and IVF cycles were divided into four seasons according to the date of the beginning of ovulation induction, which can better assess the impact of seasonal factors on ovarian response, embryo development, and pregnancy outcomes. In addition, the present study is the first to investigate the effects of seasonal variation and environmental temperature on high-quality embryo rate. However, this study also inevitably has several limitations. First, we collected retrospective data from a single center, the First Affiliated Hospital of Zhengzhou University, Henan Province; therefore, the findings cannot be applied to other regions with different climates. Additionally, indoor heating is common in some areas of Henan Province, which may have affected the study results; nevertheless, all patients were inevitably affected by the external environment. Temperature has been shown to be associated with male fertility [30–33]. In this study, we have excluded men with severe oligozoospermia or teratozoospermia, but still cannot completely rule out the effects of seasonal variation and ambient temperature on semen quality. As in previous studies, we selected the season and meteorological factors related to a specific date in the IVF process to serve as proxy for the overall environment during treatment, which may have introduced bias in the study results.

## Conclusions

This study suggests that season and ambient temperature on the date of the beginning of ovulation induction may affect embryo development and clinical pregnancy during IVF treatment. However, a multicenter study with a large sample size is required to verify the conclusion.

## Data Availability

The raw data supporting the conclusions of this article will be made available by the authors, without undue reservation.
